# Comparison of Diagnostic Accuracy of Ultrasound and Mammography in Detecting Breast Cancer in Radiographically Dense Breasts

**DOI:** 10.7759/cureus.92637

**Published:** 2025-09-18

**Authors:** Haseena Rehman, Irshad Ahmad, Saman Rashid, Muqadas Mukhtar, Aftab Ahmad Khan, Huma Khaliq

**Affiliations:** 1 General Surgery, Lady Reading Hospital Medical Teaching Institution (MTI), Peshawar, PAK; 2 Radiology, Niazi Medical and Dental College, Sargodha, PAK; 3 Radiology, Niazi Welfare Teaching Hospital, Sargodha, PAK; 4 Diagnostic Radiology, Dallah Hospital, Riyadh, SAU; 5 Nursing, Intrinsic Analytics Inc., Winnipeg, CAN; 6 Medical Oncology, Mater Hospital, Dublin, IRL; 7 Radiology, Family Health Hospital, Islamabad, PAK

**Keywords:** cancer, histopathological, implication, mammography, patients, tissue

## Abstract

Background: Breast density significantly reduces the sensitivity of mammography, complicating early cancer detection. Ultrasound has emerged as a supplemental imaging modality, especially valuable in women with dense breasts.

Objectives: This study aimed to evaluate and compare the diagnostic performance, including sensitivity, specificity, and overall accuracy, of ultrasound and mammography in detecting breast cancer among women with radiographically dense breast tissue (Breast Imaging Reporting and Data System (BI-RADS) categories C and D).

Methods: This prospective, comparative observational study was conducted at Family Health Hospital, Islamabad, Pakistan, from June 2023 to January 2024. A total of 240 female patients (mean age 48.6 ± 9.2 years; range 30-70) were enrolled. Of these, 142 (59.2%) had heterogeneously dense breasts (BI-RADS C) and 98 (40.8%) had extremely dense breasts (BI-RADS D). Mammography included standard two-view digital imaging (craniocaudal and mediolateral oblique), interpreted by radiologists using the BI-RADS classification system.

Results: Out of 240 patients, 68 (28.3%) were histologically confirmed to have breast cancer. Ultrasound showed a sensitivity of 85.3% (58/68) and specificity of 88.4% (152/172), while mammography demonstrated a sensitivity of 61.8% (42/68) and specificity of 91.9% (158/172). Sensitivity analysis by breast density revealed that mammography performed better in heterogeneously dense breasts (77.8% (14/18)) but dropped markedly in extremely dense breasts (43.8% (28/64)). Ultrasound maintained high sensitivity across both groups (86.1% (31/36) in BI-RADS C and 84.4% (27/32) in BI-RADS D). The area under the receiver operating characteristic (ROC) curve (AUC) was 0.89 for ultrasound and 0.78 for mammography.

Conclusion: It is concluded that ultrasound significantly improves breast cancer detection in women with dense breasts compared to mammography alone.

## Introduction

Primary breast cancer remains the leading type of cancer among females in the world, and its health concerns to society are immense, as its incidence rate is high, bearing the risk of metastasizing and hence affecting the quality of life. The possibility of breast cancer prognoses greatly depends on the level of cancer detection, which is why accurate diagnostic tools are essential [1]. Mammography is acknowledged by medical professionals as the necessary instrument of breast cancer screening that could reveal breast cancers at their early stages to reduce mortality in the population. Accuracy level declines boldly among women with radiologically dense breasts. Mammograms depict the ratio of fibroglandular tissue against fatty tissue that constitutes the breast density [2]. Ethnically dense breasts, which belong to Breast Imaging Reporting and Data System (BI-RADS) categories C (heterogeneously dense) and D (extremely dense), present their own set of difficulties in breast cancer screening practices [3]. High breast density is an established risk factor that causes a scenario in which 20% to 30% of sensitivity in mammograms is lost. The high breast density is a ticket to the club of risk factors since women with maximum density are at four to six times higher risk of breast cancer in comparison to those with fatty breast tissue [4].

The compromised image of dense breasts has prompted medical practitioners to apply other forms of imaging in the identification of cancer. Ultrasonic imaging has become either an aid to the mammographic examinations or an alternative to them. Mobile-controlled and automated breast ultrasound units are useful in detecting breast cancer tumors that regular mammograms fail to detect, particularly by observation of small and highly invasive lesions without having them spread to nodes [5]. Both the young female patients and patients with dense tissue benefit the most from the supplementary imaging technique, as mammography is unable to view the cases effectively [6]. The benefits of ultrasound do not free it from the technical glitches. Misunderstandings with the techniques and operator-dependent issues make this method susceptible in terms of consistency and interpretation. The detection method causes more cases of cancer to be found, but it also yields more benign test results, which causes anxiety and also more medical tests. Comparative understanding of the mammography and ultrasound when tested using the parameters of sensitivity together with the specificity and positive predictive value (PPV) and negative predictive value (NPV) is a professional decision-making process in healthcare [7].

Studies concerning such problems have yielded rather diverse results due to specific population peculiarities and imaging means and devices [8]. The evidence regarding the use of ultrasound hints at its use in supplementing mammographic screening in dense breasts and complementing mammography, showing contradictory findings concerning its supplementary role in reducing false-positive tests [9]. Medical practitioners assess the efficacy of three-dimensional tomosynthesis and contrast-enhanced mammography/MRIs and the possible application of the procedures in breast cancer diagnosis. Ultrasound is the obvious alternative because of its accessibility and being more affordable compared to other alternatives, which many medical institutions are likely to make use of in resource-limited regions [10]. 

The basic aim of the study is to evaluate and compare the diagnostic performance, including sensitivity, specificity, and overall accuracy, of ultrasound and mammography in detecting breast cancer among women with radiographically dense breast tissue (BI-RADS categories C and D).

## Materials and methods

This prospective, comparative observational study was conducted at Family Health Hospital, Islamabad, Pakistan, from June 2023 to January 2024. A total of 240 female patients were enrolled in the study. The sample size was calculated using a power analysis based on an expected difference in sensitivity between ultrasound and mammography in dense breasts, with a confidence level of 95% and a power of 80%. The study included female patients aged between 30 and 70 years who were confirmed to have radiographically dense breasts, specifically those falling under BI-RADS categories C (heterogeneously dense) and D (extremely dense) on initial mammographic evaluation. Patients with suspicious lesions were also required to consent to histopathological confirmation through biopsy or surgical excision, and only those who provided informed consent were included in the study. Women with fatty breast tissue (BI-RADS categories A and B) and those with a prior history of breast cancer or breast surgery, as well as pregnant or lactating women, were excluded from the study. Patients with incomplete imaging records or missing follow-up data were also not considered eligible.

Data collection

All enrolled patients underwent both digital mammography and high-resolution ultrasound, performed either on the same day or within a seven-day interval to maintain diagnostic consistency. Mammography included standard two-view digital imaging (craniocaudal and mediolateral oblique), interpreted by radiologists using the BI-RADS classification system. High-frequency (7.5-13 MHz) linear transducers were employed for ultrasound examinations, which systematically scanned the entire breast and axilla and focused extra attention on clinical concern areas alongside abnormal mammographic findings. Two experienced radiologists specializing in breast imaging interpreted imaging results without knowledge of each other's assessments. A third senior radiologist examined conflicting findings to achieve agreement between interpreting professionals. The research team acquired tissue diagnoses of all BI-RADS 4 or 5 lesions by performing ultrasound-guided core needle biopsies or conducting surgical excisions. The reference method for confirming malignancy or lack thereof was histopathological examination. Conservative care and six-month follow-up imaging were provided to benign-appearing lesions that received BI-RADS 2 or 3 ratings to avoid needlessly invasive procedures.

Data analysis

Data were analyzed using IBM SPSS Statistics software, version 27 (IBM Corp., Armonk, NY). The diagnostic parameters assessed for both mammography and ultrasound included sensitivity, specificity, PPV, NPV, and overall accuracy. Differences in categorical variables were assessed using the chi-square test. Receiver operating characteristic (ROC) curves were also plotted to evaluate and compare the diagnostic performance of the two modalities. A p-value of less than 0.05 was considered statistically significant in all analyses.

## Results

Data were collected from 240 patients; the mean age was 48.6 ± 9.2 years, with participants ranging from 30 to 70 years old. A majority were premenopausal (134, 55.8%), while 106 (44.2%) were postmenopausal. Notably, 38 (15.8%) had a family history of breast cancer, and 46 (19.2%) had a history of hormonal therapy use. Regarding breast density, 142 (59.2%) were classified as BI-RADS C and 98 (40.8%) as BI-RADS D. Clinically, 166 (69.2%) presented with a palpable lump, 24 (10%) had nipple discharge, and 50 (20.8%) were asymptomatic at the time of imaging (Table 1).

**Table 1:** Demographic and Baseline Characteristics of the Patients Data are presented as n (%) for categorical variables and Mean ± SD for continuous variables. BI-RADS: Breast Imaging Reporting and Data System

Characteristic	Value
Mean Age (Years)	48.6 ± 9.2
Premenopausal	134 (55.8%)
Postmenopausal	106 (44.2%)
Family History of Breast Cancer	38 (15.8%)
Hormonal Therapy Use	46 (19.2%)
BI-RADS C (Heterogeneously Dense)	142 (59.2%)
BI-RADS D (Extremely Dense)	98 (40.8%)
Palpable Lump	166 (69.2%)
Nipple Discharge	24 (10.0%)
Asymptomatic	50 (20.8%)

Mammography correctly identified 42 true positive cases and 158 true negatives, but it missed 26 cancers (false negatives) and produced 14 false positives. This resulted in a sensitivity of 61.8% and a specificity of 91.9%. The PPV was 75.0%, the NPV was 85.8%, and the overall diagnostic accuracy stood at 83.3%. In contrast, ultrasound detected a higher number of true positives (58) and maintained 152 true negatives, while misclassifying 10 cancers as false negatives and producing 20 false positives. Its diagnostic indices were stronger, with a sensitivity of 85.3% and specificity of 88.4%. The PPV was 74.4%, and the NPV was 93.8%, yielding an overall accuracy of 87.1% (Table 2).

**Table 2:** Comparison of Diagnostic Accuracy Data are presented as n (%) for categorical values. Sensitivity, specificity, PPV, NPV, and accuracy are expressed as percentages (%).

Modality	True Positive	False Negative	True Negative	False Positive	Sensitivity (%)	Specificity (%)	Positive Predictive Value (PPV, %)	Negative Predictive Value (NPV, %)	Accuracy (%)
Mammography	42	26	158	14	61.8	91.9	75	85.8	83.3
Ultrasound	58	10	152	20	85.3	88.4	74.4	93.8	87.1

**Figure 1:**
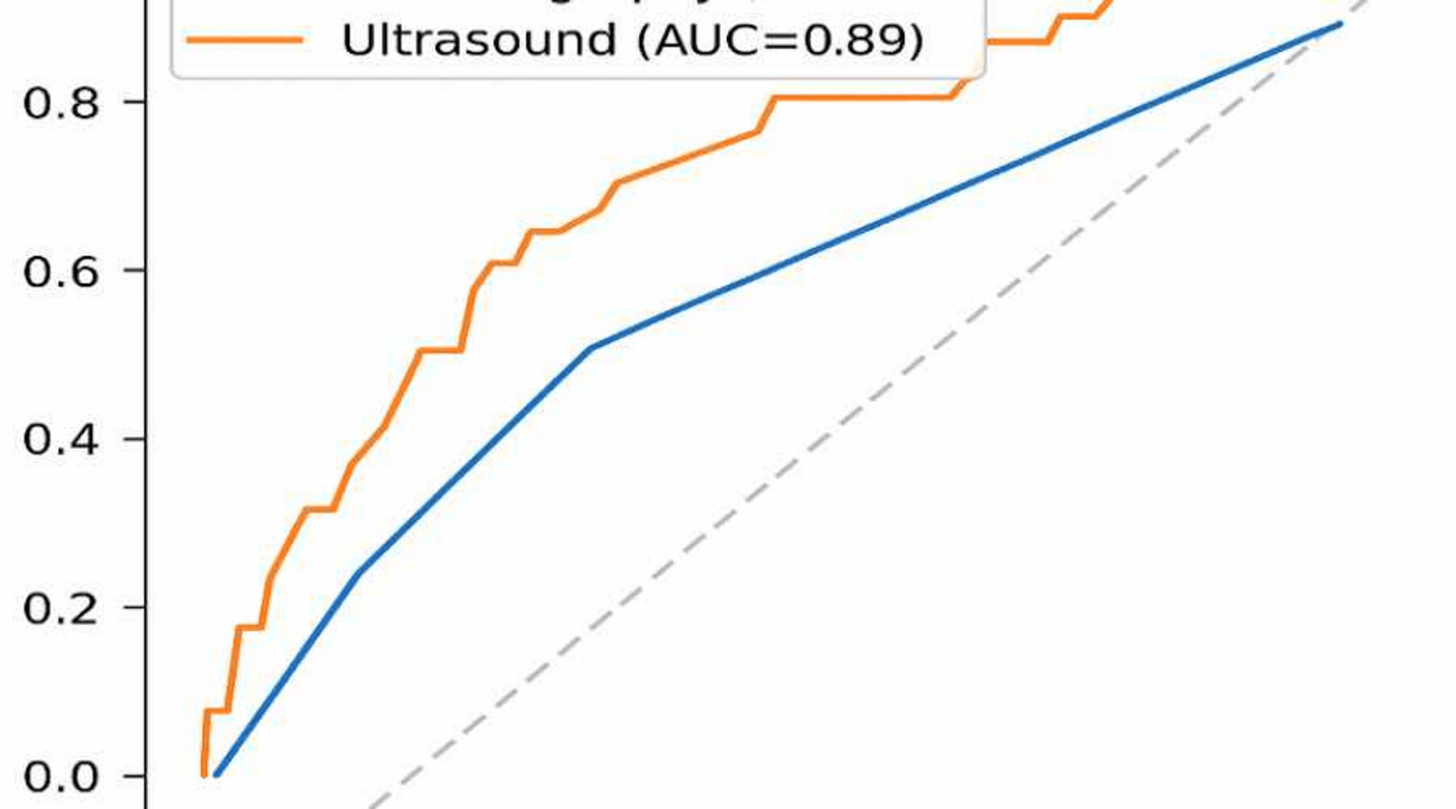
Receiver Operating Characteristic (ROC) Curve for Sensitivity and Specificity Area under the curve (AUC) is provided for each modality. Statistical significance threshold: p < 0.05.

Sensitivity analysis by breast density showed that mammography had a sensitivity of 77.8% in heterogeneously dense breasts (BI-RADS C), which declined to 43.8% in extremely dense breasts (BI-RADS D). Ultrasound maintained relatively high sensitivity in both categories, with 86.1% in BI-RADS C and 84.4% in BI-RADS D (Table 3, Figure 1).

**Table 3:** Subgroup Sensitivity by Breast Density Data are presented as percentages (%) for sensitivity within each BI-RADS category. BI-RADS: Breast Imaging Reporting and Data System

Breast Density	Modality	Sensitivity (%)
BI-RADS C (Heterogeneously Dense)	Mammography	77.8%
BI-RADS D (Extremely Dense)	Mammography	43.8%
BI-RADS C (Heterogeneously Dense)	Ultrasound	86.1%
BI-RADS D (Extremely Dense)	Ultrasound	84.4%

Out of the total 240 patients, 142 (59.2%) were classified as having heterogeneously dense breasts (BI-RADS C), while 98 patients (40.8%) had extremely dense breasts (BI-RADS D) (Table 4).

**Table 4:** BI-RADS Category Distribution Data are presented as n (%). Statistical significance was set at p < 0.05. BI-RADS: Breast Imaging Reporting and Data System

BI-RADS Category	Number of Patients	Percentage (%)
Category C (Heterogeneously Dense)	142	59.2%
Category D (Extremely Dense)	98	40.8%

**Figure 2:**
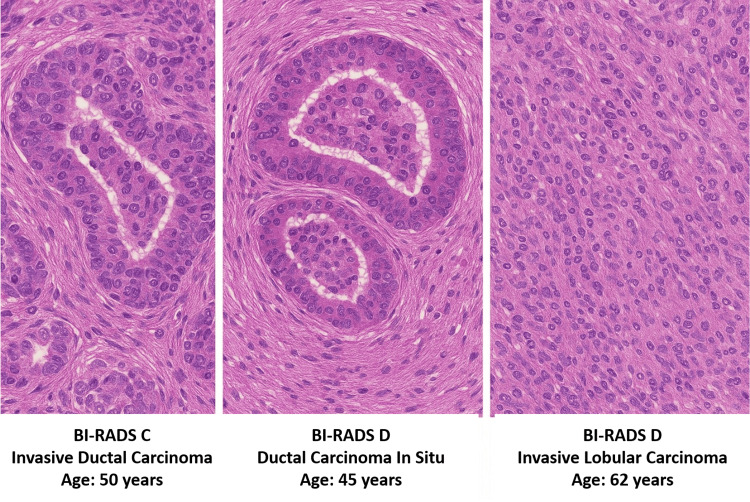
Histopathological Sections From Three Breast Cancer Patients With Dense Breast Tissue The first (BI-RADS C) illustrates invasive ductal carcinoma in a 50-year-old patient; the second (BI-RADS D) shows ductal carcinoma in situ in a 45-year-old patient; and the third (BI-RADS D) depicts invasive lobular carcinoma in a 62-year-old patient. Each case highlights distinct cellular patterns correlating with different histological subtypes. BI-RADS: Breast Imaging Reporting and Data System

Among the 68 confirmed malignant cases in the study, invasive ductal carcinoma (IDC) was the most common histopathological subtype, accounting for 52 cases (76.5%). This was followed by invasive lobular carcinoma (ILC) in 10 cases (14.7%) and ductal carcinoma in situ (DCIS) in six cases (8.8%) (Table 5, Figure 2).

**Table 5:** Histopathological Subtypes of Malignancy Data are presented as n (%).

Histopathological Subtype	Number of Cases	Percentage of Malignancies (%)
Invasive Ductal Carcinoma (IDC)	52	76.5%
Invasive Lobular Carcinoma (ILC)	10	14.7%
Ductal Carcinoma In Situ (DCIS)	6	8.8%

## Discussion

Results of this research are in support of these two points that emphasize the difficulty in diagnosing breast cancer that arises due to the denseness of the respective tissue, and hence how an additional imaging procedure may emerge as a desirable add-on to the imaging process in enhancing the percentage of cancer cases detected. Of the 240 women who had radiographically dense breasts, ultrasound had significantly higher sensitivity (85.3%) than mammography (61.8%), which proves the competence of ultrasound as a complementary tool to dense breast imaging. This coincides with other past studies, such as the American College of Radiology Imaging Network's (ACRIN) ACRIN 6666 study, which demonstrated that the incorporation of an ultrasound into mammography was able to detect more cancers, especially when used on women with dense breasts with negative mammograms [11]. An analysis of the examination results demonstrated that mammography maintained an extremely high rate of specificity at 91.9% but was inaccurate in diagnosing BI-RADS D patients, with the sensitivity rate standing only at 43.8%. The screening challenge of mammographic imaging can be manifested in dense tissues because the radiopaque glandular structures cause image obscurations, which conceal the masses. Failure to observe the microcalcification and architectural abnormalities is still one of the advantages of mammography since it is not visible in ultrasound [12].

Evidence reported that ultrasound presented exceptional levels of sensitivity and, at the same tim,e registered an NPV of 93.8% in eliminating malignant growths among women with dense breasts [13]. Ultrasound screening of breast cancer led to an increase in the correlated positive outcomes (specificity = 88.4%), a factor, however, that led to more benign tissue biopsies. This small loss in performance is useful to clinical practice due to the necessity to not miss early cancer diagnoses in high-risk groups. The effect on the response of the research shows that ultrasound is qualified in the detection of the abnormalities in the breast in the dense types of breasts that extend from the heterogeneous (BI-RADS C) to the extremely dense breasts (BI-RADS D) [14]. The sensitivity of ultrasound remained unchanged across the population groups under the study, although the mammographic sensitivity scores went down in people with dense breast tissues [15-17]. The results of the study show that special screening methods according to the breasts' density are needed to provide evidence supporting the recommendations of the American College of Radiology (ACR) and European Society of Breast Imaging (EUSOBI) concerning the addition of images to dense breast patients [18].

The histopathological correlation was confirmed in confirming the IDC dominance coupled with the ILC and DCIS. These data support the clinical importance of early and precise diagnosis since microinvasive cancers in their early stages are likely to be smalland might not present in a mammogram; however, they will in an ultrasound [19-20]. Major strengths of our work are prospective design, uniformity of imaging protocols, and histopathological validation of all suspicious results. Nevertheless, several limitations should be noted. Ultrasound is operator dependent, and there may be an element of variability in the detection rates, but we tried to reduce this by the use of experienced radiologists and consensus reading. Moreover, whereas biopsy confirmed questionable results, there was a conservatively noninvasive follow-up of a subgroup of low-suspicion cases, which would bring an element of verification bias. This study has certain limitations that should be acknowledged when interpreting the results. First, the study was conducted at a single center, which may limit the generalizability of the findings to broader populations or different healthcare settings. Second, although efforts were made to minimize observer variability by involving experienced radiologists and using consensus reading, ultrasound remains inherently operator-dependent, and performance may differ across institutions with varying expertise. Third, while all BI-RADS 4 and 5 lesions underwent histopathological confirmation, low-suspicion (BI-RADS 2 and 3) lesions were followed conservatively, which introduces a potential verification bias. Fourth, the follow-up period for BI-RADS 2 and 3 lesions was limited to six months, and the absence of longer-term surveillance raises the possibility that late-developing malignancies could have been missed, potentially underestimating false negatives.

## Conclusions

It is concluded that ultrasound is a significantly more sensitive diagnostic modality than mammography in detecting breast cancer in women with radiographically dense breasts, particularly in BI-RADS D classifications. While mammography retains high specificity and remains valuable for detecting microcalcifications and subtle structural abnormalities, its reduced sensitivity in dense tissue limits its effectiveness as a standalone tool. In contrast, ultrasound not only enhances the detection of malignancies that are mammographically occult but also offers a high negative predictive value, making it an effective adjunct in breast cancer screening protocols for dense breasts.
